# Detection of SARS-CoV-2 in saliva: implications for specimen transport and storage

**DOI:** 10.1099/jmm.0.001285

**Published:** 2020-12-03

**Authors:** Eloise Williams, Nicole Isles, Brian Chong, Katherine Bond, Yano Yoga, Julian Druce, Mike Catton, Susan A. Ballard, Benjamin P. Howden, Deborah A. Williamson

**Affiliations:** ^1^​ Department of Microbiology, Royal Melbourne Hospital, Melbourne, Australia; ^2^​ Microbiological Diagnostic Unit, Department of Microbiology and Immunology, The University of Melbourne at The Peter Doherty Institute for Infection and Immunity, Melbourne, Australia; ^3^​ Victorian Infectious Diseases Reference Laboratory at The Peter Doherty Institute for Infection and Immunity, Melbourne, Australia

**Keywords:** coronavirus disease, COVID-19, molecular microbiology, saliva, SARS-CoV-2

## Abstract

Saliva has recently been proposed as a suitable specimen for the diagnosis of severe acute respiratory syndrome coronavirus 2 (SARS-CoV-2). Use of saliva as a diagnostic specimen may present opportunities for SARS-CoV-2 reverse transcription polymerase chain reaction (RT-PCR) testing in remote and low-resource settings. Determining the stability of SARS-CoV-2 RNA in saliva over time is an important step in determining optimal storage and transport times. We undertook an *in vitro* study to assess whether SARS-CoV-2 could be detected in contrived saliva samples. The contrived saliva samples comprised 10 ml pooled saliva spiked with gamma-irradiated SARS-CoV-2 to achieve a concentration of 2.58×10^4^ copies ml SARS-CoV-2, which was subsequently divided into 2 ml aliquots comprising: (i) neat saliva; and a 1 : 1 dilution with (ii) normal saline; (iii) viral transport media, and (iv) liquid Amies medium. Contrived samples were made in quadruplicate, with two samples of each stored at either: (i) room temperature or (ii) 4 °C. SARS-CoV-2 was detected in all SARS-CoV-2 spiked samples at time point 0, day 1, 3 and 7 at both storage temperatures using the N gene RT-PCR assay and time point 0, day 1 and day 7 using the Xpert Xpress SARS-CoV-2 (Cepheid, Sunnyvale, USA) RT-PCR assay. The ability to detect SARS-CoV-2 in saliva over a 1 week period is an important finding that presents further opportunities for saliva testing as a diagnostic specimen for the diagnosis of SARS-CoV-2.

Saliva has recently been proposed as a suitable diagnostic specimen for the diagnosis of severe acute respiratory syndrome coronavirus 2 (SARS-CoV-2) [1–3]. Saliva has a number of advantages compared to nasopharyngeal swab (NPS) sampling including (i) reduction of risk to healthcare workers during the close contact and potential aerosol generation involved in collection of NPS, (ii) reduction of patient discomfort involved in NPS collection and (iii) absence of requirements for specialized consumables and trained healthcare workers. Compared to NPS specimens, the sensitivity of saliva for detection of severe acute respiratory syndrome coronavirus 2 (SARS-CoV-2) has ranged between 78–100 % in the published literature [1–8]. Studies have also demonstrated the feasibility and performance of saliva sampling for SARS-COV-2 as diagnostic specimens in the ambulatory setting [1, 3].

Use of saliva as a diagnostic specimen may present opportunities for SARS-CoV-2 reverse transcription polymerase chain reaction (RT-PCR) testing in remote and low-resource settings, as well as allowing scalable population-level screening. Determining the stability of SARS-CoV-2 RNA in saliva over time is an important step in determining optimal storage and transport times but to date, there are no studies describing the stability of SARS-CoV-2 RNA in saliva. Here, we undertook an *in vitro* study to assess whether SARS-CoV-2 could be detected in saliva by RT-PCR using three different transport media at different temperatures over a 7 day period.

A ‘mock’ sample matrix was constructed, consisting of 10 ml of pooled saliva that tested negative to SARS-CoV-2 using an in-house E gene RT-PCR using previously published primers [4]. This pooled sample was spiked with gamma-irradiated SARS-CoV-2 to achieve a final concentration of 2.58×10^4^ copies ml of SARS-CoV-2 strain VIC001 [9], and subsequently divided into 2 ml aliquots comprising: (i) neat saliva; and a 1 : 1 dilution with (ii) normal saline; (iii) viral transport media (University of Melbourne Media Preparation Unit, Melbourne, Australia; product no. 2512), and (iv) liquid Amies medium (University of Melbourne Media Preparation Unit, Melbourne, Australia; product no. 2162).

In total, 200 ul aliquots of each contrived sample were made in quadruplicate, with two samples of each stored at either: (i) room temperature (average temperature over the study period of 16 °C) or (ii) 4 °C. At time point 0, day 1, 3 and 7, aliquots underwent RNA extraction using the QIAamp 96 Virus QIAcube HT Kit (QIAGEN, Hilden, Germany) and were eluted in 60 ul. Reverse transcription was performed using the BioLine SensiFAST cDNA kit (Bioline, London, UK) as previously described [9]. cDNA underwent PCR using an in-house real-time assay using previously described primers targeting the SARS-CoV-2 N gene [10]. In addition, 300 ul aliquots of each contrived sample were tested at day 0, 1 and 7 using the Xpert Xpress SARS-CoV-2 assay (Cepheid, Sunnyvale, USA) on the GeneXpert Infinity platform (Cepheid, Sunnyvale, USA), which targets the E and N2 genes [3].

SARS-CoV-2 was detected in all SARS-CoV-2 spiked samples at all time points, and at both storage temperatures using the N gene assay (Table 1) and was not detected in 18 negative control samples comprising six replicates of normal saline, liquid Amies and viral transport media at timepoint 0. In addition, SARS-CoV-2 was detected in neat saliva, and saliva combined with various transport media (Table 1). One replicate (1/64; 1.5 %) did not have SARS-CoV-2 detected; this was an aliquot of saliva mixed 1 : 1 with liquid Amies and stored at room temperature for 7 days. Moreover, SARS-CoV-2 was detected using the Xpert Xpress SARS-CoV-2 (Cepheid, Sunnyvale, USA) by both the E and N2 gene targets at day 0, 1 and 7 at both storage temperatures, and in all transport conditions (Table S1, available in the online version of this article).

**Table 1.** SARS-CoV-2 N gene RT-PCR results for saliva samples combined with various transport media and exposed to various storage conditions over a 7 day period

Storage condition/time point	Mean (variance) N gene Cycle threshold value			
	Neat saliva	Normal saline	Liquid Amies	VTM
**Room temperature**				
Time zero	34.5 (0.5)	32.0 (0.0)	33.0 (0.0)	33.0 (2.0)
24 hours	37.0 (2.0)	38.0 (0.0)	35.5 (4.5)	36.5 (0.5)
72 hours	38.0 (2.0)	37.0 (0.0)	36.5 (0.5)	34.0 (0.0)
168 hours	36.5 (4.5)	38.0 (2.0)	36.0*	34.5 (0.5)
**4 °C**				
Time zero	34.5 (0.5)	33.0 (0.0)	34.0 (0.0)	33.0 (0.0)
24 hours	36.5 (0.5)	36.0 (0.0)	34.0 (0.0)	35.0 (0.0)
72 hours	35.0 (0.0)	34.5 (0.5)	34.5 (0.5)	34.5 (0.5)
168 hours	37.0 (2.0)	35 (2.0)	37.0 (2.0)	35.5 (0.5)

*One replicate of gamma-irradiated SARS-CoV-2 spiked saliva diluted 1 : 1 with liquid Amies not detected on N gene RT-PCR after storage at room temperature for 168 hours; VTM, viral transport media.

The variability in sensitivity of SARS-CoV-2 detection by RT-PCR in saliva specimens reported in the published literature likely reflects various patient, sampling and analytical factors. The majority of studies show a higher rate of detection of SARS-CoV-2 by RT-PCR in NPS specimens [2–5, 8], however some studies have demonstrated a higher rate of detection in saliva specimens compared to paired NPS specimens [7, 11], or detection of SARS-CoV-2 in a number of saliva samples with paired negative NPS [1, 3, 8]. There is biological plausibility that samples collected from the oral cavity may be an appropriate specimen type, with a recent study demonstrating high ACE2 receptor expression in the epithelial cells of oral mucosa and the base of the tongue [12]. Defining the ideal specimen collection, transport and processing methods for saliva samples is key to optimizing the role of saliva samples in the diagnosis of SARS-CoV-2.

The ability to detect SARS-CoV-2 in saliva over a 1 week period is an important finding that presents further opportunities for saliva testing as an initial screening test in hard-to-reach populations where there may be few alternative options. Further studies are required to assess the reproducibility of SARS-CoV-2 RNA detection in saliva samples over this period in tropical climates with higher room temperature and humidity and with RT-PCR targets using other regions of the SARS-CoV-2 genome.

Coupled with the use of a ‘near care’ technology such as the Xpert Xpress SARS-CoV-2, this may significantly increase access to SARS-CoV-2 testing in some settings [3]. These include: (i) remote locations with limited healthcare providers, where samples may require significant transit time; (ii) low resource settings in which sampling consumables and personal protective equipment are limited and (iii) vulnerable populations in which nasopharyngeal swab collection is not an acceptable sampling method. Repeat sampling with a nasopharyngeal swab may then be reserved as a second-line test for those with an ongoing high clinical index of suspicion.

## Supplementary Data

Supplementary material 1
