# Supplementary material for: Detection of SARS-CoV-2 in saliva: implications for specimen transport and storage
Source: J Med Microbiol. 2020 Dec 3;70(2):001285. doi: 10.1099/jmm.0.001285 (PMC8131016; doi:10.1099/jmm.0.001285)
Supplement: Supplementary material 1 [file jmm-70-285-s001.pdf]

Supplementary Table 1. Xpert Xpress SARS-CoV-2 RT-PCR results for pooled saliva samples combined with various transport media and exposed to various storage conditions over a seven-day period.

**Storage condition / time point**	**E gene Ct value**				**N2 gene Ct value**			
	**Neat saliva**	**Normal saline**	**Liquid Amies**	**VTM**	**Neat saliva**	**Normal saline**	**Liquid Amies**	**VTM**
**Room temperature**								
Time zero	29.5.	29.1	28.1	28.3	32.1	32.7	30.9	31.6
24 hours	27.9	28.4	28.3	28.4	30.5	30.9	31.6	31.2
168 hours	28.6	28.4	28.4	28.8	31.9	30.8	31.1	31.2
**4°C**								
Time zero	27.6	28.3	28.4	28.1	30.7	30.8	31.2	31.4
24 hours	28.3	29.0	28.3	28.4	31.4	32.4	31.4	31.3
168 hours	29.5	28.4	28.6	28.7	31.9	31.6	31.5	31.5

Ct, cycle threshold; VTM, viral transport media.
